# The Dysregulation of Essential Fatty Acid (EFA) Metabolism May Be a Factor in the Pathogenesis of Sepsis

**DOI:** 10.3390/medicina60060934

**Published:** 2024-06-03

**Authors:** Undurti N. Das

**Affiliations:** 1 UND Life Sciences, 2221 NW 5th St., Battle Ground, WA 98604, USA; undurti@hotmail.com; Tel.: +1-508-904-5376; 2 Department of Biotechnology, Indian Institute of Technology-Hyderabad, Sangareddy 502285, India; 3 Department of Medicine, Omega Hospitals, Gachibowli, Hyderabad 500032, India

**Keywords:** sepsis, hyperinflammation, immunosuppression, essential fatty acids, eicosanoids, corticosteroids

## Abstract

I propose that a deficiency of essential fatty acids (EFAs) and an alteration in their (EFAs) metabolism could be a major factor in the pathogenesis of sepsis and sepsis-related mortality. The failure of corticosteroids, anti-TNF-α, and anti-interleukin-6 monoclonal antibodies can be attributed to this altered EFA metabolism in sepsis. Vitamin C; folic acid; and vitamin B1, B6, and B12 serve as co-factors necessary for the activity of desaturase enzymes that are the rate-limiting steps in the metabolism of EFAs. The altered metabolism of EFAs results in an imbalance in the production and activities of pro- and anti-inflammatory eicosanoids and cytokines resulting in both hyperimmune and hypoimmune responses seen in sepsis. This implies that restoring the metabolism of EFAs to normal may form a newer therapeutic approach both in the prevention and management of sepsis and other critical illnesses.

## 1. Introduction

Despite many years of research, the molecular mechanisms of the cause of morbidity and mortality of sepsis remained an enigma. Usually, sepsis is triggered by a systemic infection, resulting in dysregulated immune response. Sepsis is the major cause of death in intensive care units worldwide. Sepsis occurs because of complex interactions among invading organisms, tissue damage, and alterations in pro- and anti-inflammatory mechanisms at various stages of the disease that result in excessive inflammation, immunosuppression, and potentially long-term immune disorders [1,2]. Immunosuppression due to sepsis enhances the chance of re-infection. It is known that antibiotics, fluid resuscitation, and organ support therapy have limited impacts on the prognosis of sepsis. Efforts made to suppress excessive inflammation by corticosteroids and neutralize increased TNF-α and IL-6 cytokines have been ineffective [3]. The potential role of essential fatty acids (EFAs) and their metabolites in sepsis has largely been neglected but may hold the key to developing newer preventive and therapeutic approaches to sepsis. The important role of vitamins, including vitamin C; folic acid; vitamins B1, B6, B12; corticosteroids; and cytokines, in the metabolism of EFAs may explain the relative efficacy or inefficiency of various therapeutic strategies employed. I propose that co-administration of EFAs in combination with corticosteroids, vitamins, and anti-cytokine therapy may be of benefit in sepsis. 

## 2. Metabolism of Essential Fatty Acids

**Figure 1:**
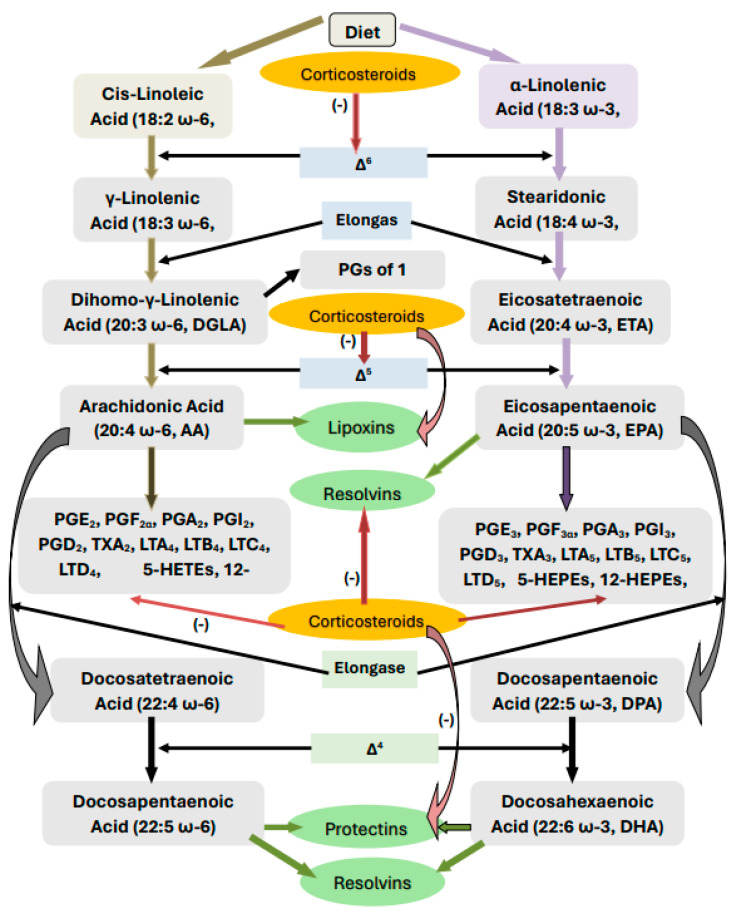
Scheme showing metabolism of EFAs and the effect of corticosteroids on EFAs metabolism. **−** indicates inhibition of formation/action. Green refers to useful actions. While brick color refers to harmful actions.

Dietary linoleic acid (c-LA, 18:2 n-6) and alpha-linolenic acid (ALA, 18:3 n-3) are essential fatty acids that are not formed in the body and, hence, must be obtained from diet. EFAs are essential for the integrity of skin and immune response and form precursors of several pro- and anti-inflammatory eicosanoids. EFAs are metabolized by enzymes desaturases (Δ^6^ and Δ^5^) and elongases to form their long-chain metabolites: gamma-linolenic acid (GLA, 18:3 n-6), dihomo-GLA (DGLA, 20:3 n-6), and arachidonic acid (AA, 20:4 n-6) from LA and eicosapentaenoic acid (EPA, 20:5 n-3) and docosahexaenoic acid (DHA, 22:6 n-3) from ALA. All these compounds are polyunsaturated fatty acids (PUFAs: LA, GLA, DGLA, AA, ALA, EPA, and DHA). DGLA is the precursor of prostaglandin E1 (PGE1), a potent platelet anti-aggregator; AA is the precursor of two series prostaglandins (PGs), thromboxanes (TXs), and four series leukotrienes (LTs) that have pro-inflammatory actions except for PGI2 (prostacyclin) that has platelet anti-aggregator and vasodilator and both pro- and anti-inflammatory actions. AA is also the precursor of anti-inflammatory, vasodilator, and platelet anti-aggregator molecule lipoxin A4 (LXA4). EPA is the precursor of 3-series PGs, TXs, and 5-series LTs that also possess pro-inflammatory actions but are much less potent compared to those derived from AA. In addition, anti-inflammatory compounds resolvins (RSVs) of the E series are derived from EPA. DHA does not form any PGs, LTs, and TXs but gives rise to potent anti-inflammatory compounds, resolvins of the D series, protectins, and maresins. Thus, PUFAs form precursors to both pro- and anti-inflammatory compounds. The balance between these pro- and anti-inflammatory compounds ultimately determines whether the inflammatory process continues or inflammation resolution occurs, leading to wound healing ([4,5,6,7,8,9,10] and Figure 1).

For the normal activity of desaturases, which are the rate-limiting steps in the metabolism of EFAs, several co-factors are needed. Vitamins, including folic acid, B1, B6, B12, and magnesium, are some of the co-factors needed for the normal activity of desaturases, whereas vitamin C is necessary for the conversion of DGLA to PGE1. High glucose, a lack of insulin, cholesterol, and ethanol suppress the activity of desaturases (Δ^6^ > Δ^6^). Various viruses, cytokines, interleukin-6 (IL-6), tumour necrosis factor-α (TNF-α), and some gut microbiota can suppress the activities of desaturases [4]. The conversion of AA to various eicosanoids is triggered by the activation of cyclo-oxygenase-2 (COX-2) enzyme, whereas its conversion of LXA4 needs lipoxygenase (LOX) enzyme. There are three types of LOX enzymes, namely 5-LOX, 12-LOX, and 15-LOX. Both COX and LOX enzymes are activated by various pro-inflammatory factors, including but not limited to infections, injury, and chemicals. The release of AA and other PUFAs from the cell membrane phospholipid moiety is induced by the activation of phospholipase A2 (PLA2) by various stimuli. Thus, the formation of various eicosanoids (PGs, LTs, TXs, LXA4, resolvins, protectins, and maresins) from their respective precursors needs COX-2 and LOX enzymes. Thus, an adequate formation of pro- and anti-inflammatory eicosanoids needs a timely availability of adequate amounts of EFAs from the diet and of various co-factors that are needed for optimum function of desaturases. Deficiency in LA and ALA and/or the inappropriate activation of any desaturases, PLA2, COX, and LOX enzymes, or a deficiency of the co-factors outlined above can result in inappropriate inflammation, delay in wound healing, and the continuation of inflammation. 

## 3. EFAs and Cytokines

It is noteworthy that almost all PUFAs and PGs, LXA4, resolvins protectins, and maresins suppress the production and action of pro-inflammatory IL-6, TNF-α, and HMGB1 (high-mobility group box-1). PGE2, though a pro-inflammatory molecule, also suppresses the production of IL-6 and TNF-α, suggesting the existence of a freed back regulatory system between PUFAs, eicosanoids, and cytokines. In contrast, IL-6 and TNF-α augment PGE2 and LT production, which may perpetuate the inflammatory process. LXA4, resolvins, protectins, and maresins are potent suppressors of IL-6 and TNF-α. This close interaction(s) among PUFAs, eicosanoids, and cytokines seems to be crucial in the onset, continuation, and resolution of inflammation [4,5,6,7,8,9,10].

Paradoxically, pro-inflammatory cytokines (IL-6 and TNF-α), despite their ability to enhance PLA2 activity, have been shown to inhibit the actions of desaturases [11] and, thus, induce deficiency of DGLA, AA EPA, and DHA. This can result in reduced formation of PGs, LTs, TXs, LXA4, resolvins, protectins, and maresins, which may have an adverse effect on the inflammatory process and its resolution. 

This intricate but complex network of events may explain the difficulties encountered in inducing the resolution of the inflammatory process, especially in sepsis. It is important that inflammation-inducing events such as the formation of cytokines and eicosanoids and the synthesis and release of anti-inflammatory LXA4, resolvins, protectins, and maresins are required in an orderly and sequential manner not only to induce the much-needed inflammatory process but also its timely resolution. 

## 4. Corticosteroids and EFAs 

**Figure 2:**
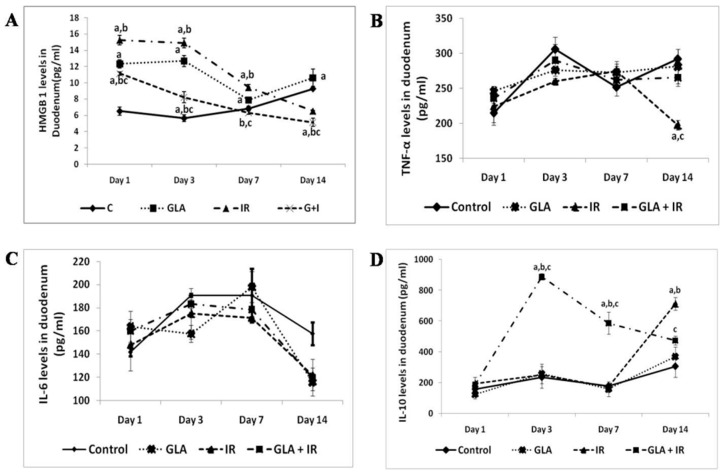

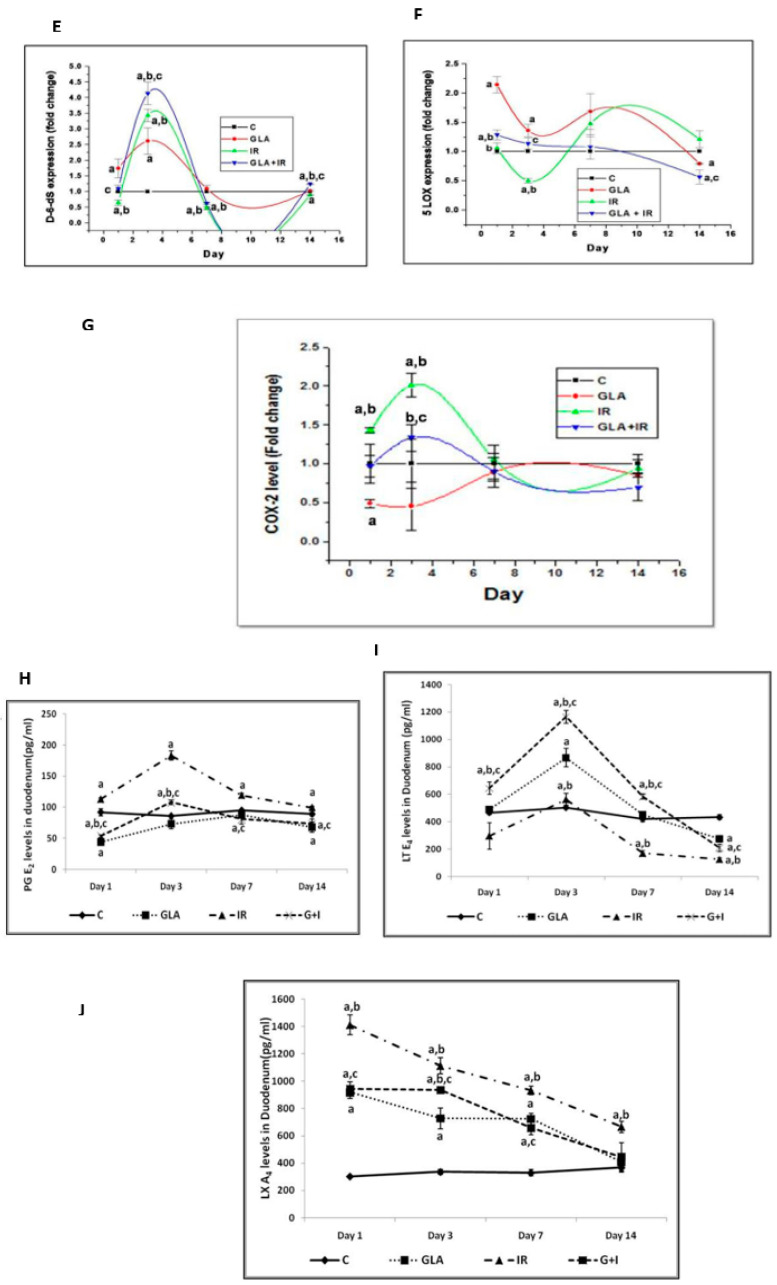

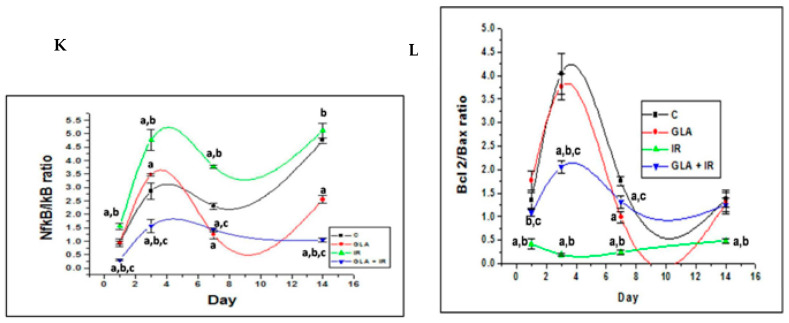
(**A**–**D**) Duodenal concentrations of cytokines HMGB1 (**A**), TNF-α (**B**), IL-6 (**C**), and IL-10 (**D**) by ELISA method. Mice were pre-treated with 100 µg/kg GLA at 48, 24, and 1 h prior to total-body irradiation of 7.5 Gy (at 1 Gy/min). Duodenum samples were obtained on day 1, day 3, day 7, and day 14 post-irradiation. All values are expressed as mean ± SEM (*n* = 3). Statistical significance was calculated using *t*-tests, and *p*-values < 0.05 are represented by a, b, c when compared to control, GLA, and irradiation, respectively. C, control; GLA, gamma-linolenic acid; IR, irradiation (7.5 Gy). (**E**–**G**) Effect of GLA and irradiation on genes associated with PUFA metabolism in duodenum tissue on day 1, day 3, day 7, and day 14. Values (*n* = 3) expressed as mean ± SEM. a, b, c were significant (*p* < 0.01) when compared with control, GLA, and irradiation, respectively. (**H**–**J**) Estimation of PUFA metabolite PG E2, LTE4, and LXA4 levels. Mice were pre-treated with 100 μg/kg GLA at 48, 24, and 1 h prior to irradiation and were subjected to total-body irradiation of 7.5 Gy (at 1 Gy/min). Duodenum samples were obtained on day 1, day 3, day 7, and day 14 post-irradiation. All the values are expressed as mean ± SEM (*n* = 3). Statistical significance was calculated using *t*-tests, and *p*-values < 0.001 are represented by a, b, c when compared to control, GLA, and IR, respectively. C, control; GLA, gamma-linolenic acid; IR, irradiation (7.5 Gy). (**K**,**L**): Effect of GLA and radiation on the expression of NF-kB/IkB in duodenal tissue of mice on days 1, 3, 7, and 14. All values (*n* = 3) are expressed as mean ± SEM. a, b, c were significant (*p* < 0.01) when compared with control, GLA, and IR, respectively. C, control; GLA, gamma-linolenic acid; IR, irradiation (7.5 Gy). Genes associated with Apoptotic Pathway Radiation are known to cause apoptosis/cell death. Hence, we studied their effect on the expression of genes associated with apoptosis and cell survival. Our results show that IR caused a significant (*p* < 0.01) decrease in the Bcl-2/Bax ratio on all days (1, 3, 7, and 14) of the study compared to the control. This indicates a significant degree of cell death in the duodenum tissues. In contrast to this, GLA + IR-treated mice showed a significantly (*p* < 0.01) higher Bcl-2/Bax ratio, indicating improved survival of cells against irradiation-induced insult at all time points. These data (this figure) are taken from reference [28].

Corticosteroids are potent anti-inflammatory compounds. Our previous studies revealed that corticosteroids inhibit the activities of PLA2, desaturases, COX-2, and LOX enzymes [12,13]. As a result, the release of (i) DGLA/AA/EPA/DHA may be insufficient from the cell membrane lipid pool, (ii) the conversion of dietary EFAs to their respective long-chain metabolites will be inadequate, and (iii) the conversion of DGLA/AA/EPA/DHA to their respective PGs, LTs, TXs, LXA4, resolvin, protectins, and maresins will be defective. Thus, initially, corticosteroids suppress inflammation by blocking the formation of PGs, LTs, and TXs, producing their anti-inflammatory action. However, their continued use results in a state of PUFAs deficiency (low amounts of GLA/DGLA/AA/EPA/DHA are formed from LA and ALA). As a result, an inadequate formation of LXA4, resolvins, protectins, and maresins (due to the deficiency of their precursors and suppression of COX-2 and LOX enzymes) occurs, resulting in the inappropriate resolution of inflammation and defective wound healing. Furthermore, we and others showed that AA and LXA4, resolvins, protectins, and maresins have potent anti-inflammatory, anti-obesity, anti-diabetic, and anti-hypertensive actions and prevent insulin resistance (EPA and DHA and their metabolites also have similar action but are less potent compared to AA and LXA4) [4,14,15,16,17,18,19,20,21,22,23,24,25,26,27,28]. This suggests that abnormalities in EFA metabolism could be responsible for the Cushingoid features, insulin resistance, hyperglycemia, hypertension, poor wound healing, and excess fluid retention seen in those who are on corticosteroids for considerable time. Thus, it is anticipated that the continued use of corticosteroids will result in a state of EFAs deficiency and profound alterations in their metabolism and the formation of their metabolites (see Figure 1). In addition, the corticosteroids-induced suppression of IL-6 and TNF-α will also affect EFAs metabolism. The presence of adequate amounts of PGE2 is needed to trigger the production of LXA4 to shift the inflammatory process from a pro-inflammatory state to a resolution phase. This implies that the induction of an adequate degree of inflammation is needed to trigger its resolution process by shifting AA conversion from pro-inflammatory PGE2 to an anti-inflammatory LXA4 state. This intricate and complex role of various cytokines, PUFAs, and their metabolites in inflammation and its resolution is evident from our recent study with radiation in experimental animals that showed this complex behaviour of molecules (see Figure 2). Our study revealed that suppressing the radiation-induced inflammatory process can be restored to normal by the timely administration of GLA that bypasses the inhibitory action of radiation on desaturases [28]. A similar approach is needed to suppress the sepsis process as well. 

## 5. Antimicrobial Action of EFAs

Inhaled air contains various microbes that need to be eliminated by an efficient surveillance system in the lungs. Alveolar macrophages protect us from these microbes by releasing LA, GLA, and AA that have potent anti-microbial action [29,30,31,32,33,34]; NK cells; and cytotoxic tumour lymphocytes, such as CTL (cytotoxic lymphocyte) cells, lymphokine-activated killer cells, dendritic cells, and leukocytes, which have been shown to kill various microbes even in the absence of perforin and granzyme by activating soluble PLA2 (sPLA2) that induce the release of AA and other fatty acids [32]. Unsaturated fatty acids have been shown to form an important constituent of cytolytic granules of CTL, NK, and γδT cells [33] that not only induce apoptosis of tumour cells but also produce their antimicrobial action. The importance of PLA2/PLD lies in the fact that these enzymes are needed to induce the release of unsaturated fatty acids from the cell membrane lipid pool of various cells, including various immunocytes. 

## 6. M1 and M2 Macrophages and EFAs

**Figure 3:**
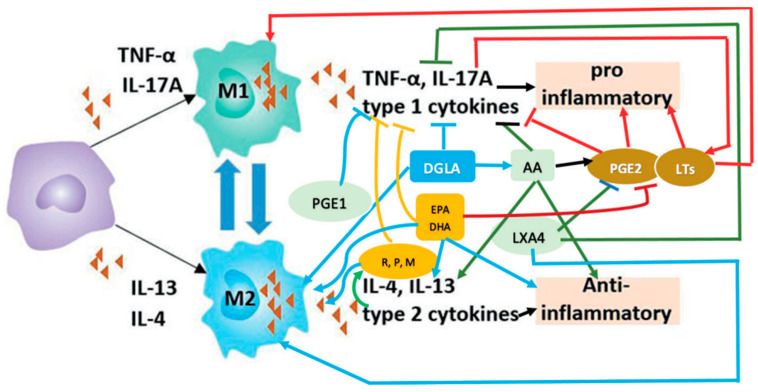
Scheme showing actions of M1 and M2 macrophages and cytokines produced by them. DGLA, AA, EPA, and DHA are anti-inflammatory in nature and suppress the production of TNF-α, IL-2, and IL-1 and facilitate M2 macrophages generation. PGE1 from DGLA and LXA4 from AA, resolvins (R) of E series from EPA and E series from DHA. Protectins (P) and maresins (M) from DHA are anti-inflammatory. Resolvins, protectins, and maresins suppress TNF, IL-1, IL-2. PGE2 production. Leukotrienes of B4, D4, and E4 series from AA are pro-inflammatory. Leukotrienes of 5 series are from EPA and are less pro-inflammatory compared to leukotrienes of 4 series. LXA4, resolvins, protectins, and maresins inhibit PGE2 and LTs production. EPA and DHA inhibit PGE2 production. This figure is taken from reference [34].

Macrophages play an important role in the induction and resolution of inflammation and wound healing. They release several soluble mediators to perform these functions. Macrophages are of two types: M1 and M2. M1 types are pro-inflammatory, whereas M2 are anti-inflammatory in nature. M1 kills the invading microbes and clears the debris, whereas M2 are needed to resolve inflammation and restore homeostasis (see Figure 3). 

M1 macrophages elaborate PGE2 and LTs (leukotrienes), and in turn, these eicosanoids facilitate their development (M1 macrophages). In contrast, anti-inflammatory cytokines IL-4 and IL-13 and lipoxin A4 (LXA4) (derived from AA), resolvins (from eicosapentaenoic acid (EPA)), and resolvins, protectins, and maresins (derived from DHA)} (reviewed in [5,31,34,35,36,37,38,39]) facilitate the development of M2 macrophages, and in turn, all these anti-inflammatory molecules are secreted by M2 macrophages. These facts suggest that the availability of adequate amounts of AA, EPA, and DHA is essential for the formation and secretion of anti-inflammatory molecules in a timely fashion and in adequate amounts to suppress inappropriate inflammation and restore homeostasis. TNF-α and IL-6 suppress the activities of desaturases, which are crucial for the conversion of dietary LA and ALA to AA, EPA, and DHA (see Figure 1) and, as a result, can cause GLA, DGLA, AA, EPA, and DHA deficiency. This leads to a reduced generation of LXA4, resolvins, protectins, and maresins due to their respective precursor deficiency. Since lipoxins, resolvins, protectins, and maresins suppress IL-6 and TNF-α formation and have anti-inflammatory action, it is suggested that excess generation of IL-6 and TNF-α that results in cytokine storm in severe COVID-19, ARDS (acute respiratory distress), sepsis, and other acute conditions could be due to a deficiency of GLA/DGLA/AA/EPA/DHA and their anti-inflammatory metabolites. 

## 7. PGE2 and LXA4 Interact to Initiate Resolution of Inflammation

AA is the precursor of both PGE2 and LXA4. Despite the general belief that PGE2 is a pro-inflammatory molecule, recent studies revealed that it also has anti-inflammatory action. PGE2 secreted by M1 macrophages is also a facilitator of their (M1 macrophages) development. In contrast, M2 macrophages secrete LXA4 and LXA4, in turn facilitating their (M2 macrophages) development. Once PGE2 concentrations reach an optimum level, they could trigger LXA4 generation and simultaneously suppress LTB4 production by their ability to enhance 5- and 15-lipoxygenase expression. This triggers the suppression of the pro-inflammatory process and initiates the anti-inflammatory events that ultimately result in the resolution of inflammation and restoration of homeostasis. The exact mechanism by which the metabolism of AA is redirected to generate LXA4 in the place of PGE2 is not clear. However, it could be attributed to the biphasic release of AA from the cell membrane lipid pool (reviewed in [5]).

The potential anti-inflammatory and beneficial actions of PGE2 are supported by the recent report that 15-PGDH–(15-prostaglandin dehydrogenase, a prostaglandin degrading enzyme)-deficient mice not only showed a twofold increase in the levels of PGE2 in bone marrow, colon, and liver tissues but also showed augmented hematopoietic capacity, rapid liver regeneration, and enhanced recovery of neutrophils, platelets, and erythrocytes [40]) following tissues damage. Bone marrow-derived mesenchymal stem cells (MSCs) have been shown to reduce the severity of acute lung injury by secreting LXA4 and downregulating TNF-α, IL-6, IL8, and IFN-γ formation [41]. Obesity, type 2 diabetes mellitus, hypertension, and coronary heart disease and elderly subjects have reduced plasma levels of AA/EPA/DHA ([42], see Table 1) that, in turn, may lead to enhanced production of pro-inflammatory cytokines due to the absence of negative feedback regulation exerted by these lipids (including PGE2) on IL-6 and TNF-α production. These observations suggest that a delicate balance needs to be maintained between pro- and anti-inflammatory cytokines, eicosanoids, and their precursors to produce adequate inflammation in response to injury, infection, and other adverse events and, at the same time, initiate timely tissue repair and restore homeostasis. 

## 8. Brain–Body Immune Regulation—A Role for Bioactive Lipids

In this context, the role of the brain in the regulation of immune response and inflammation needs a brief discussion. It is well recognised that the brain has a regulatory role in the functions of various organs. Recently, it was shown that neurons in the brain stem modulate the course of inflammatory responses [43]. It was reported that silencing the neurons in the caudal nucleus of the solitary tract in the brainstem enhances inflammation, whereas their activation suppresses the inflammation that parallels the changes in the levels of pro- and anti-inflammatory cytokines that depend on the activation of two discrete non-overlapping populations of vagal sensory neurons. The activation of the excitatory (glutamatergic) neurons effectively suppressed inflammation. In contrast, dopamine beta-hydroxylase-expressing neurons in the brainstem suppressed pro-inflammatory cytokines and enhanced the anti-inflammatory IL-10 levels. Dopamine beta-hydroxylase converts dopamine to norepinephrine and epinephrine, which have pro-inflammatory actions [44]. Previously, it was shown that acetylcholine, the principal vagal neurotransmitter, enhances the formation of anti-inflammatory LXA4 [45]. The human brain is rich in AA and DHA. Since vagal fibers have a regulatory role in inflammation, it is likely that AA and DHA and their respective pro- and anti-inflammatory eicosanoids regulate the pro- and anti-inflammatory responses. It is suggested that infection/injury-induced pro-inflammatory events (enhanced production of PGE2) will be sensed by the enteric nerves and conveyed to the brain via pro-inflammatory vagal fibres to silence the neurons in the caudal nucleus of the solitary tract in the brainstem, leading to systemic inflammation. This inflammatory stimuli augments AA and DHA release (due to the activation of PLA2) that leads to increased formation of pro-inflammatory PGs, LTs, and TXs both locally and systemically to produce inflammation. Once the concentrations of PGE2 (and other pro-inflammatory eicosanoids) reach their peaks, they induce the formation of LXA4, resolvins, protectins, and maresin that activate (glutamatergic) neurons resulting in the suppression of systemic inflammation that is mediated via anti-inflammatory vagal fibres. Thus, the vagus may participate both in pro- and anti-inflammatory events by its actions on the caudal nucleus of the solitary tract in the brainstem that contains two discrete non-overlapping populations of vagal sensory neurons. These results are further confirmed by the recent report that chronic vagus nerve stimulation (VNS) altered mRNA and protein expression, induced the apoptosis of B cells, and impaired plasma cell differentiation. It was noted that α7 and α9 nicotinic acetylcholine receptors (nAChRs) are present in B cells, and their stimulation decreased TNF production [46]. Furthermore, it was found that LXA4, resolvins, protectins, and maresins may have antimicrobial action and, thus, are of benefit in sepsis and other illnesses [47,48,49,50]. Such an interaction between anti-inflammatory eicosanoids (LXA4, resolvins, protectins, and maresins) may also explain the role of gut microbiota in inflammatory diseases, including sepsis [51,52,53,54,55] since, previously, it was shown that PUFAs alter gut microbiota [56,57,58]. In this gut–brain–immunocyte–cytokine–EFA axis, there could be a role for serotonin and other neurotransmitters that are known to interact with acetylcholine. These results suggest that infusion of appropriate amounts of GLA/AA/EPA/DHA (especially AA/DHA) into the caudal nucleus of the solitary tract in the brainstem directly using a stereotaxic procedure or intraventricularly may form a novel method of regulating inflammation. 

In addition, patients with sepsis have altered EFAs metabolism ([59]; see Table 2) in the form of decreased concentrations of GLA, DGLA, AA, ALA, and EPA compared to a normal control. Even those with bacterial pneumonia, rheumatoid arthritis (RA), and lupus (systemic lupus erythematosus, SLE), who are more prone to develop sepsis, have dysregulated EFAs metabolism [59]. Thus, EFAs metabolism is altered in many inflammatory conditions, suggesting a critical role for bioactive lipids in these diseases. Hence, employing corticosteroids in these conditions may further worsen the altered EFAs metabolism, which may explain why steroids were of no significant benefit in sepsis and other critical illnesses. This implies that efforts need to be made to restore EFAs metabolism to normal and ensure the formation of adequate amounts of various pro- and anti-inflammatory cytokines and eicosanoids in a timely fashion to overcome the side effects of corticosteroids by their timely use. It is also important to administer not only various EFAs (especially GLA/AA/EPA/DHA) but also co-factors that are needed for their metabolism, such as vitamins C, B1, B6, B12; folic acid; magnesium; insulin; and adequate amounts of protein. In view of the pulsatile and day-to-day variation in the levels of cytokines and eicosanoids (see Figure 2), it is worthwhile to measure them as frequently as possible (at least once in 24 h) as shown and proposed previously [36,37,60,61]. Furthermore, this concept explains why the administration of vitamin C, B1, B6, and/or B12 alone gave variable results [62,63,64]. Studies performed in animal models of sepsis showed that LXA4, protectins, and resolvins are of benefit, though more in-depth studies are needed, especially in patients with sepsis [60,61,65,66,67,68,69,70,71,72,73]. 

It is noteworthy that a recent study reported an IL-1β/STAT5 signalling in T_H_17 cells may mediate steroid resistance [74]. It was shown that IL-1β signalling in T_H_17 cells promotes a steroid-resistant transcriptional program that overrides dexamethasone-induced transcriptional responses, sustaining the expression of pro-inflammatory cytokines. It was found that interleukin-1 receptor (IL-1R) blockade made the experiment sensitive to dexamethasone (Dex) treatment. This is because IL-1β induced a signal transducer and activator of transcription 5 (STAT5)-mediated steroid-resistant state in T_H_17 cells that led to the promotion of inflammatory cytokine production and suppressed dexamethasone-induced anti-inflammatory genes. This was confirmed by the T_H_17-specific deletion of STAT5 that resulted in the ablation of the IL-1β–induced steroid-resistant transcriptional program and made the animals sensitive to dexamethasone treatment. These findings are noteworthy since they imply that IL-1β–STAT5 signalling in T_H_17 cells is responsible for corticosteroid resistance [74] in those with sepsis and other critical illnesses.

In this context, it is interesting to know that several unsaturated fatty acids (including GLA, EPA, and DHA) and their anti-inflammatory metabolites, such as LXA4, resolvins, protectins, and maresins, suppress IL-1β and IL-17 production and inhibit STATs [75,76,77,78,79,80,81,82,83,84,85]. These results are in accordance with the potent anti-inflammatory actions of these bioactive lipids, further supporting their potential use in sepsis and other critical illnesses. 

## 9. Conclusions

It is evident from the preceding discussion that corticosteroid use can result in an EFAs deficiency state that, in turn, leads to the reduced formation of PGE1/PGE2, LXA4, resolvins, protectins, and maresins that are not only anti-inflammatory compounds but are needed for tissue repair, regeneration, and wound healing. This may explain why corticosteroids are ineffective in the prevention and management of sepsis. 

Since LXA4, resolvins, protectins, and maresins have very short half-lives and are unstable, it is suggested that oral or parenteral administration of GLA/AA/EPA/DHA may be tested in the prevention and management of sepsis. In view of the gut–brain–immunocyte–cytokine–EFAs axis, it may be worthwhile to study whether GLA/AA/EPA/DHA can be given intraventricularly (a procedure that is common in neurosurgical practice and safe). It is important to develop stable synthetic analogues of LXA4, resolvins, protectins, and maresins for potential use in the future, not only for sepsis but also for other inflammatory conditions. 

## Figures and Tables

**Table 1:** Plasma phospholipid concentrations of EFAs and their long-chain metabolites in patients with hypertension (HTN), coronary heart disease (CHD), type 2 diabetes mellitus (type 2 DM), and diabetic nephropathy (DN). These data are taken from reference [42]. All values are expressed as mean ± SD. * *p* < 0.05 compared to control (normal).

Fatty Acid	Control	HTN	CHD	T2DM	DN
18:2 n-6 (LA)	18.6 ± 3.1	14.5 ± 3.1 *	17.8 ± 5.0	13.9 ± 5.3	15.1 ± 3.1
18:3 n-6 (GLA)	0.14 ± 0.1	0.4 ± 0.3 *	0.1 ± 0.1 *	0.2 ± 0.3	0.1 ± 0.2
20:3 n-6 (DGLA)	3.4 ± 1.0	3.1 ± 0.9	2.7 ± 1.1	1.7 ± 1.0 *	2.0 ± 0.8 *
20:4 n-6 (AA)	9.4 ± 1.8	7.8 ± 2.0 *	7.0 ± 2.1 *	4.6 ± 1.8 *	6.6 ± 2.6 *
22:5 n-6	0.7 ± 0.4	0.4 ± 0.4 *	1.0 ± 0.9	2.1 ± 0.6 *	1.3 ± 0.5 *
20:4 n-6/18-2 n-6	0.51	0.54	0.39	0.33	0.43
18:3 n-3 (ALA)	0.2 ± 0.1	0.4 ± 0.2 *	0.3 ± 0.5	0.1 ± 0.2 *	0.1 ± 0.1
20:5 n-3 (EPA)	0.4 ± 0.4	0.6 ± 0.6	0.1 ± 0.2 *	0.3 ± 0.3	0.2 ± 0.3
22:5 n-3	0.5 ± 0.2	0.4 ± 0.5	0.3 ± 0.3 *	1.6 ± 1.3	1.7 ± 1.1
22:6 n-3 (DHA)	1.4 ± 0.5	1.2 ± 0.6	0.8 ± 0.4 *	0.5 ± 0.4 *	0.5 ± 0.3 *
20:5 n-3/18:3 n-3	1.8	1.39	0.41	3.2	4.0

**Table 2:** Fatty acid analysis of the plasma PL (phospholipid) fraction in patients with pneumonia, septicemia, RA, and lupus. This table is from reference [59].

Fatty Acid	Control (n = 10)	Pneumonia (n = 12)	Septicemia (n = 14)	RA (n = 12)	SLE (n = 5)
16:0	24.8 ± 3.4	32.5 ± 3.6	26.95 ± 4.1	30.2 ± 3.0	32.0 ± 3.75
18:0	23.3 ± 4.1	21.4 ± 7.1	24.58 ± 6.0	19.0 ± 6.1	14.6 ± 5.82
18:1 n-9	13.1 ± 2.3	15.6 ± 3.2	16.5 ± 3.3 *	14.8 ± 2.1	16.0 ± 2.76
18:2 n-6	17.7 ± 3.1	14.2 ± 0.3 *	16.3 ± 2.4	17.5 ± 2.7	20.8 ± 2.2
18:3 n-6	0.13 ± 0.09	0.13 ± 0.08	0.04 ± 0.05 *	0.02 ± 0.04 **	0.01 ± 0.01 **
20:3 n-6	3.2 ± 0.79	1.5 ± 0.4 *	0.46 ± 0.54 *	2.5 ± 0.58	2.12 ± 0.52
20:4 n-6	8.8 ± 2.0	5.1 ± 0.4 *	5.8 ± 1.6 *	9.5 ± 2.2	8.93 ± 2.0
22:4 n-6	0.42 ± 0.23	0.8 ± 0.9	0.34 ± 0.28	0.26 ± 0.37 **	0.18 ± 0.18 **
22:5 n-6	0.73 ± 0.55	0.45 ± 0.63	1.5 ± 1.02 *	0.6 ± 0.7	0.8 ± 1.0
18:3 n-6/18:2 n-6	0.007	0.0092	0.002	0.001	0.004
20:4 n-6/18:2 n-6	0.35	0.36	0.5	0.54	0.44
20:4 n-6/20:3 n-6	4.01	3.4	2.75	3.8	4.2
18:3 n-3	0.27 ± 0.12	0.09 ± 0.04 *	0.16 ± 0.11 *	0.12 ± 0.16 *	0.1 ± 0.1 *
20:5 n-3	0.25 ± 0.26	0.23 ± 0.24	0.01 ± 0.01 *	0.05 ± 0.14 **	0.04 ± 0.04 **
22:5 n-3	0.54 ± 0.16	0.44 ± 0.53	0.29 ± 0.12 *	0.69 ± 0.05	0.21 ± 0.35 *
22:6 n-3	1.43 ± 0.43	0.54 ± 0.43 *	1.2 ± 1.14	0.62 ± 0.56 *	0.88 ± 0.75 *
20:5 n-3/18:3 n-3	0.92	1.55	0.06	0.41	0.40

All values ae expressed as mean ± S.D. ** *p* < 0.001 compared to control; * *p* < 0.05 compared to control. 18:3 n-6/18:2 n-6 ratio denotes the activity of ∆^6^ desaturase. 20:4 n-6/20:3 n-6 ratio denotes the activity of ∆^5^ desaturase.
